# Biochemical Bone Markers During the Transition Period Are Not Influenced by Parenteral Treatment With a High Dose of Cholecalciferol but Can Predict Milk Fever in Dairy Cows

**DOI:** 10.3389/fvets.2020.591324

**Published:** 2021-02-05

**Authors:** Jože Starič, Jaka Jakob Hodnik

**Affiliations:** Clinic for Reproduction and Large Animals – Section for Ruminants, Veterinary Faculty, University of Ljubljana, Ljubljana, Slovenia

**Keywords:** cattle, hypocalcaemia, estradiol, bone alkaline phosphatase, vitamin D_3_, C-terminal telopeptide of type I collagen, bone turnover, screening

## Abstract

Despite being studied extensively, there are still many knowledge gaps in milk fever prevention and it is still a prevalent disease. Various interventions have been used in its prevention; however, none has proven to be entirely effective. The study aimed to assess the effectiveness of high dose vitamin D_3_ parenteral (intramuscularly) administration and the mechanism of its action by studying blood minerals and biochemical bone markers. Further, we assessed the potential of biochemical bone markers, measured in the close-up dry period, as predictors of clinical milk fever after calving. The study was conducted on 56 high yielding, clinically healthy dairy cows, before their 4th or higher lactation. They were divided into three groups based on season (summer and winter) and administration (vitamin D). The winter group was considered as the control group. Cows (*n* = 13) were parenterally administered a single dose of 10 million IU of vitamin D_3_ (DUPHAFRAL® D3) ranging between 10 and 2 days before calving (median = 3 days). Each cow was blood sampled once during four sampling period ranges: ~1 month before calving, 10 to 2 days before calving, 12–48 h after calving and 10–20 days after calving. The samples were analyzed for blood minerals, bone specific alkaline phosphatase (bALP) and C-terminal telopeptide of type I collagen (CTx), alkaline phosphatase, and estradiol. Values were compared between samplings and groups. A receiver operating characteristic (ROC) analysis and logistic regression were used to assess the diagnostic accuracy of biochemical bone markers in predicting milk fever. In this study high dose vitamin D_3_ supplementation did not statistically reduced the incidence of milk fever (milk fever incidences were 15.4, 39.1, and 25% in the vitamin D, winter and summer groups, respectively). A significant effect of vitamin D_3_ administration on blood minerals or biochemical bone markers was not found at any sampling. We found that the use of biochemical bone markers in the close-up dry period to predict clinical milk fever was applicable only in the winter (housed) group. The area under the curve (AUC) for bALP was 0.804 and 0.846 for CTx using ROC analysis. The bALP curve had the best ratio at the cut-off point 13.85 U/L with 90% sensitivity and 64.3% specificity. While CTx had the ratio of 90% sensitivity and 78.6% specificity at the cut-off point 0.149 ng/mL. Close-up dry dairy cows with CTx ≥0.121 ng/mL had a 3.8 times higher chance of succumbing to milk fever. We were unable to prove that high dose vitamin D_3_ parenteral administration is a viable technique for milk fever prevention. Biochemical bone markers are a promising tool for predicting milk fever; however, further studies are needed to confirm their clinical use.

## Introduction

High producing dairy cows often suffer from clinical or subclinical hypocalcaemia because of a sudden high demand for calcium (Ca) in the days around calving (1, 2). This is the result of high amount of Ca rich colostrum and milk production coupled with the inability to activate calcium homeostasis mechanisms fast enough to compensate the loss of Ca (3). Milk fever is a disease with a high direct and indirect economic impact (1, 4, 5). Consequently, many studies tackle prevention and control of hypocalcaemia in dairy cattle, however, to date no simple and effective method has been found and the disease is still prevalent in dairy cattle herds globally. Furthermore, a screening test for the identification of cows, which are at higher risk of hypocalcaemia and need intervention to prevent it does not exist. Currently, the most common prevention strategies for milk fever are anion salt supplementation, feeding of low calcium or low available calcium diet before calving, and vitamin D supplementation (1). All mentioned interventions are effective to some degree, but none entirely.

We still do not understand the key mechanisms involved in milk fever prevention (6). Possible mechanisms include higher Ca uptake from the intestine, increased bone resorption, or alternatively, reduced Ca uptake by other tissues and processes (1). Vitamin D supplementation has had varied effectiveness in the past, which can be explained, to some degree, by the use of different supplementation protocols but the underlying mechanism is still unknown (7–9).

The objective of this study was to evaluate the effect of a single high-dose parenteral administration of vitamin D_3_, between 10 and 2 days before calving and season on blood biochemical bone markers, blood minerals, total alkaline phosphatase, estradiol, and milk yield in standard lactation during the transition period compared to untreated cows during the winter housed period. Few studies have investigated the effect of different milk fever prevention methods on selected parameters to such extent (6, 9–11). The hypothesis was that vitamin D_3_ administration would increase bone resorption, raise blood mineral content, reduce the incidence of milk fever and increase milk production compared to untreated groups. Further, the study aimed to investigate the association of biochemical bone markers in the close-up dry period and clinical milk fever.

## Methods

### Animals and Experimental Groups

The study was performed in a large Slovenian commercial dairy farm with 205 dairy cows and accompanying young stock (*n* = 175) of the Holstein-Friesian breed, free of bovine viral diarrhea (BVD) and infectious bovine rhinotracheitis (IBR). The production group was housed in a free-stall barn with concrete slatted floors and cubicles. The calving stall was a tie-stall type barn with rubber mats, additionally littered with sawdust. Young stock were kept in a separate barn. Cows were milked twice a day in a 2 × 10 herringbone style milking parlor. Cows were dried-off about 60 days before calving. In summer, cows were on pasture the whole day (far-off dry cows) or had daily access to pasture from their stall (close-up dry cows, fresh cows).

**Figure 1:**
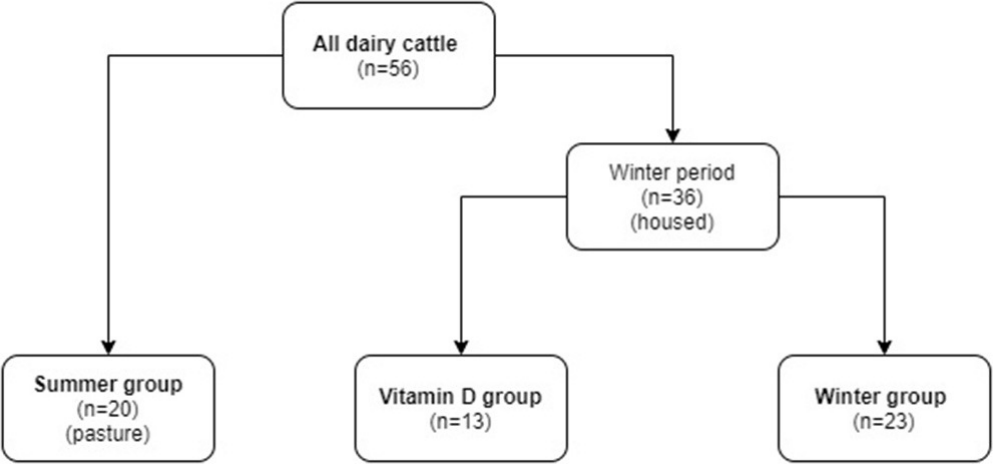
The allocation of dairy cows to study groups.

Clinically healthy (*n* = 56) high yielding cows (mean 8,700 kg milk per standard lactation) that were in the middle of the dry period expected to give birth to at least their 4th calf, were enrolled in the study. The health status of enrolled cows, which were between 5 and 11 years old, was confirmed by clinical examination and complete blood count with inclusion where values were within the reference range for dairy cows. To investigate the effects of season the study was conducted in two parts. The first part (summer) was between 19th April and 11th September at which time animals had daily access to pasture. The second (winter) was between 26th October and 4th April when animals were housed in the barn all the time, without access to direct sunlight. Animals were randomly assigned to three groups depending on season and vitamin D_3_ administration (Figure 1). Cows were considered as having milk fever if they exhibited clinical signs of milk fever (recumbency after parturition) and responded to milk fever treatment (the cows stood up after treatment with 2 g of Ca/100 kg body weight intravenously). The diagnosis was confirmed by measurement of blood total calcium (tCa) <1.6 mmol/L (12). Cows were managed on the farm as they would be routinely.

### Diet

Cows were fed a total mixed ration (TMR) twice a day after morning and afternoon milking (except the summer dry cows) throughout the study. The composition and the nutrient content of the rations and pasture, maize silage, grass silage, and hay can be seen in Tables 1, 2. The pasture was sampled from different locations of the pasture to collect 1 kg of forage for analysis. Care was taken that plants that the cows avoid were not included in the sample. In winter, far-off dry cows were fed the dry cow TMR *ad libitum*. In summer, they were on pasture and also feed hay with a vitamin mix. The close-up cows were fed 11–12 kg DM of close up TMR per cow per day with access to *ad libitum* hay in winter and pasture in summer. Fresh cows received 15 kg DM of fresh cow TMR per cow per day. They also had access to hay in winter and pasture for 2–3 h per day in summer.

**Table 1:** Composition of the food ration in kg for each stage of production with consumption of minerals and Net Energy for Lactation (NEL).

**Feedstuff (in kg)**	**Summer dry cows pasture**	**Winter dry cows TMR**	**Close up TMR**	**Fresh cows TMR**
Pasture	40	/	/	/
Straw	/	3	0.2	0.2
Grass silage	/	12	7	8
Maize silage	/	7	14	16
Soybean meal	/	/	0.3	0.4
Anti-ketosis concentrate with propylene glycol (Emona, Slovenia)	/	/	0.5	1
K Mix concentrate (Emona, Slovenia)	/	/	1.5	2
Vitamin mix (Emona, Slovenia)	0.05 (Rumisal for dry cows)	0.1 (Rumisal for dry cows)	0.1 (Vitamix top)	0.15 (Vitamix top)
+ Hay	4	2	2	3
DM intake (kg)	10.7	11.3	11.9	15
**Nutrient and energy content**				
NEL (MJ)	61.3	61.75	77.55	97.8
Crude fiber (g)	3056.5	3162.5	2380.8	2935
Crude protein (g)	1571	1039.5	1437.2	1856.2
Ca (g)	66.25	80.17	90.69	120.08
P (g)	42.74	32.48	37.52	49.25
Mg (g)	34.19	38.15	31.07	40.57
Na (g)	16.03	11.79	20.19	28.75
K (g)	195.57	160.90	146.61	183.26
Cl (g)	121.83	69.62	69.9	95.52
S (g)	28.85	22.49	23.54	30.99
DCAD (mEq/kg DM)^*^	+40	+270	+320	+200

*Anti-ketosis concentrate (NEL 9 MJ/kg, crude protein 14 %, digestible crude protein 11%, Ca 0.8%, P 0.4%, Na 0.25%, Mg 0.25%)*.

*K Mix concentrate (NEL 6.6 MJ/kg, Crude protein 26%, digestible crude protein 21.5%, Ca 1.2%, P 0.6%, Na 0.45%, Mg 0.3%)*.

*Rumisal for dry cows (3% Ca, 8% P, 7% Mg, 125,000 IU/kg Vitamin D_3_)*.

*Vitamix Top (20% Ca, 3.5% P, 7% Na, 5% Mg, 70,000 IU Vitamin D_3_)*.

* *DCAD = (Na^+^ + K^+^) – (Cl^−^ + S^2−^) (13)*.

*The data were processed using the nutrition software developed by a Slovenian feed manufacturer Jata Emona d.o.o. (Slovenia) for their own use*.

*DM, Dry matter; NEL, Net Energy for Lactation; Ca, Calcium; P, Phosphorus; Mg, Magnesium; Na, Sodium; K, Potassium; Cl, Clor; S, Sulfur; DCAD, Dietary Cation-Anion Difference*.

**Table 2:** The nutrient content of pasture forage, maize silage, grass silage, and hay.

	**Pasture**	**Maize silage**	**Grass silage**	**Hay**
Dry matter (%)	19.8	39.9	30.4	89.3
Ash (% DM)	0.96	3.1	13.13	6.22
Crude protein (% DM)	20	6.6	13.6	/
Crude fat (% DM)	2.9	4.6	3.0	/
Crude fiber (% DM)	23.1	19.1	24.1	/
Degradable protein (g/kg)	15.4	2.96	9.6	/
Phosphorus (% DM)	0.4	0.19	0.29	0.28
Calcium (% DM)	0.61	0.18	1.28	0.6
Magnesium (% DM)	0.29	0.096	0.44	0.23
Potassium (% DM)	1.99	0.77	2.10	1.49
Sodium (% DM)	0.17	0.003	0.10	0.049

### Vitamin D_3_ Supplementation

Every third cow enrolled into the experiment in wintertime was administered 10,000,000 IU (10 mL) of vitamin D_3_ (DUPHAFRAL® D3, Fort Dodge Veterinaria, S.A., Vall de Bianya, Spain) intramuscularly (*m. semimembranosus*) between 10 and 2 days before calving (median = 3 days).

### Sampling

**Figure 2:**
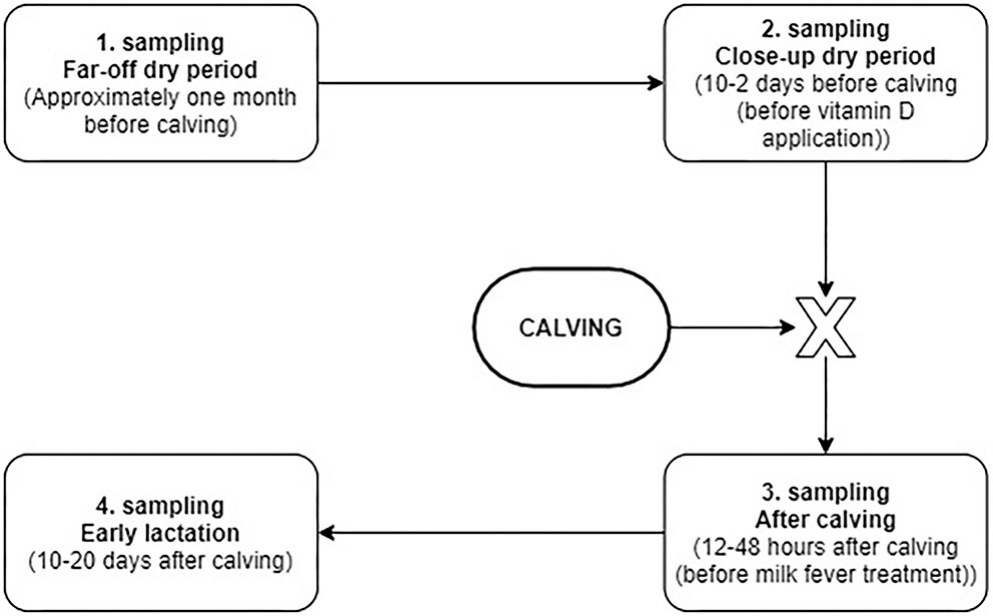
Flowchart of the blood sampling protocol.

Blood samples were taken four times according to the study protocol (Figure 2), based on metabolic profiling protocols (14). Blood was collected via venipuncture of the *v. caudalis mediana* into serum evacuated tubes (Venoject, plain silicone-coated, Terumo Europe N.V., Belgium). All samples were collected between 9 and 12 a.m. Blood was left to coagulate at room temperature. Blood serum was obtained the same day by centrifugation twice at 3,000 rpm for 10 min at room temperature and equally divided into four aliquots and frozen at −20°C until analysis.

### Analysis

Samples were analyzed for concentration of minerals [total calcium (tCa), inorganic phosphates (iP), and magnesium (Mg)], alkaline phosphatase (ALP), bones specific alkaline phosphatase (bALP), C-terminal telopeptide of type I collagen (CTx), and 17β-estradiol.

Values of tCa, iP, Mg and ALP were determined using Calcium Colorimetric Method, Inorganic Phosphorus UV Method, Magnesium (Xylidil Blue) Colorimetric Method and ALP opt. Liquid Alkaline Phosphatase, respectively, on the RX Daytona analyser (Randox, Ireland) using commercial kits from Randox (CA3871, PH3820, MG3880, AP3802 respectively; Randox, Laboratories Ltd., United Kingdom).

bALP was analyzed using ELISA Alkphase–B kit (formally K981373, Metra, Biosystems, USA after taking over by Quidel, USA, MicroVue BAP EIA 8012). The assay uses monoclonal mouse anti bALP antibodies, tested on bALP from a human osteosarcoma cell line, which coat the microtiter plates. Five microliters of cow serum was added and incubated for 3 h then washed. The substrate was added, and the absorbance was measured using an optic reader Humareader (Human, Egypt) at 405 nm using a standardized curve after 30 min. We made the curve using six standardized concentrations of bALP: 0.0; 2.0; 20.0; 40.0; 80.0; and 140.0 U/L; respectively. The method was validated on human samples (15).

CTx was analyzed using ECLIA ELECSYS beta–CrossLaps (11972308122, Roche Diagnostics, USA) method. The electrochemiluminescent sandwich immunoassay uses two monoclonal antibodies against beta-isomerized 8-amino acid sequence (EKAHD-beta-GGR) of the beta-CTx. The sample (50 μL) was incubated with the antibodies for 9 min. Then ruthenium-labeled antibodies and streptavidin-coated paramagnetic microbeads were added. A sandwich complex was formed that binds to the beads. After an additional 9 min of incubation, the concentration of CTx was determined using the ROCHE Elecsys 1010 Immunoassay analyser (Roche Diagnostics, USA). The test was validated on human serum samples (16).

The concentrations of 17β-estradiol were analyzed using Immulite 2000—Estradiol (L2KE22(D), Siemens Medical Solutions Diagnostics, USA) with the detection range between 0.073 and 7.242 nmol/L (17). The solid phase (bead) is coated with polyclonal rabbit anti-estradiol antibodies. The liquid phase is alkaline phosphatase conjugated to estradiol. The sample and the reagent were incubated for 60 min. The bead was washed, and a chemiluminescent substrate was added. The results were measured after 5 min. The test was validated by the manufacturer.

All analyses were carried out according to the manufacturer's instructions in the Laboratory of clinical pathology, Veterinary faculty, University of Ljubljana and the General hospital Jesenice in Slovenia, both involved in international quality control schemes.

### Analysis of Milk Production

The information on milk yield, fat % and protein % was obtained from the Agricultural Institute of Slovenia, which preforms monthly milk recording on farms according to the AT4 method of the International Comittee of Animal Recording (ICAR) guidelines (18). Energy corrected milk (ECM) was calculated using the formula ECM (kg) = milk yield (kg) × (0.383 × fat (%) + 0.242 x protein (%) + 0.7832)/3.14 (19).

### Statistical Analysis

Statistical analysis was performed using R statistical software (The R Foundation for Statistical Computing, Austria). The threshold for statistical significance was set at *p* < 0.05.

To analyse differences between group means at different samplings we used a pairwise Wilcoxon rank sum test. The Bonferroni correction was used to counteract the problem of multiple comparisons. We used the Kruskal–Wallis test to assess the statistical difference in lactation number and Body Condition Score (BCS). The graphs were made using the ggplot2 package in R statistical software (20).

To determine the effects of vitamin D_3_ administration compared to the winter group we used Student's *t*-test (iP, bALP, CTx at 3rd sampling and bALP at 4th sampling) or Wilcoxon rank sum test (tCa at 3rd sampling and tCa, iP, CTx at 4th sampling).

To assess the correlations between tCa, iP, bALP, CTx, and estradiol at different samplings we used Kendall's correlation.

To determine the diagnostic use of bALP and CTx, in close-up dry cows, to predict milk fever we used linear regression. Significant results in the winter group were further analyzed using the receiver operating characteristic (ROC) curve and AUC (area under the curve) with 95% confidence intervals using the pROC package in R statistical software (21). The ROC curve was produced using the bALP and CTx concentrations in the close-up dry period and the data on the occurrence of clinical milk fever at third sampling. A best cut-off point to determine the specificity and sensitivity of bALP and CTx to predict milk fever was used. To analyse the correlation of CTx with clinical milk fever incidence we use simple logistic regression. The CTx values were separated into two groups using the median.

The Fisher's exact test to assess the difference between proportions of milk fever between experimental groups was used.

To assess the effect of group on milk yield and energy corrected milk (ECM) milk yield we used the Kruskal–Wallis test, and one-way ANOVA for fat % and protein %.

## Results

The cows enrolled in the study were healthy and comparable between groups (Table 3).

**Table 3:** Comparison of groups in far-off dry period for lactation number and body condition score (BCS).

	**Lactation**	**BCS**
Summer (pasture)	5.38 ± 1.07	3.06 ± 0.48
Vitamin D (housed)	5.46 ± 1.81	3.58 ± 0.37^*^
Winter (housed)	5.36 ± 1.56	3.37 ± 0.47
*P*-value (Kruskal–Wallis)	0.82	<0.05

*Mean ± SD*.

* *indicates a statistically significant difference between the Summer and Vitamin D groups*.

### Effect of High Dose Vitamin D_3_ Administration on Milk Fever Incidence, Milk Yield and Quality

Out of the 13 cows in the vitamin D group, only two developed milk fever (15.4%) while 9 (39.1%) out of the remaining 23 cows in the winter (housed) part of the study, developed milk fever. Five (25%) out of 20 cows developed milk fever in the summer (pasture) group. However, there was no statistically significant difference between groups using the Fisher's exact test.

**Figure 3:**
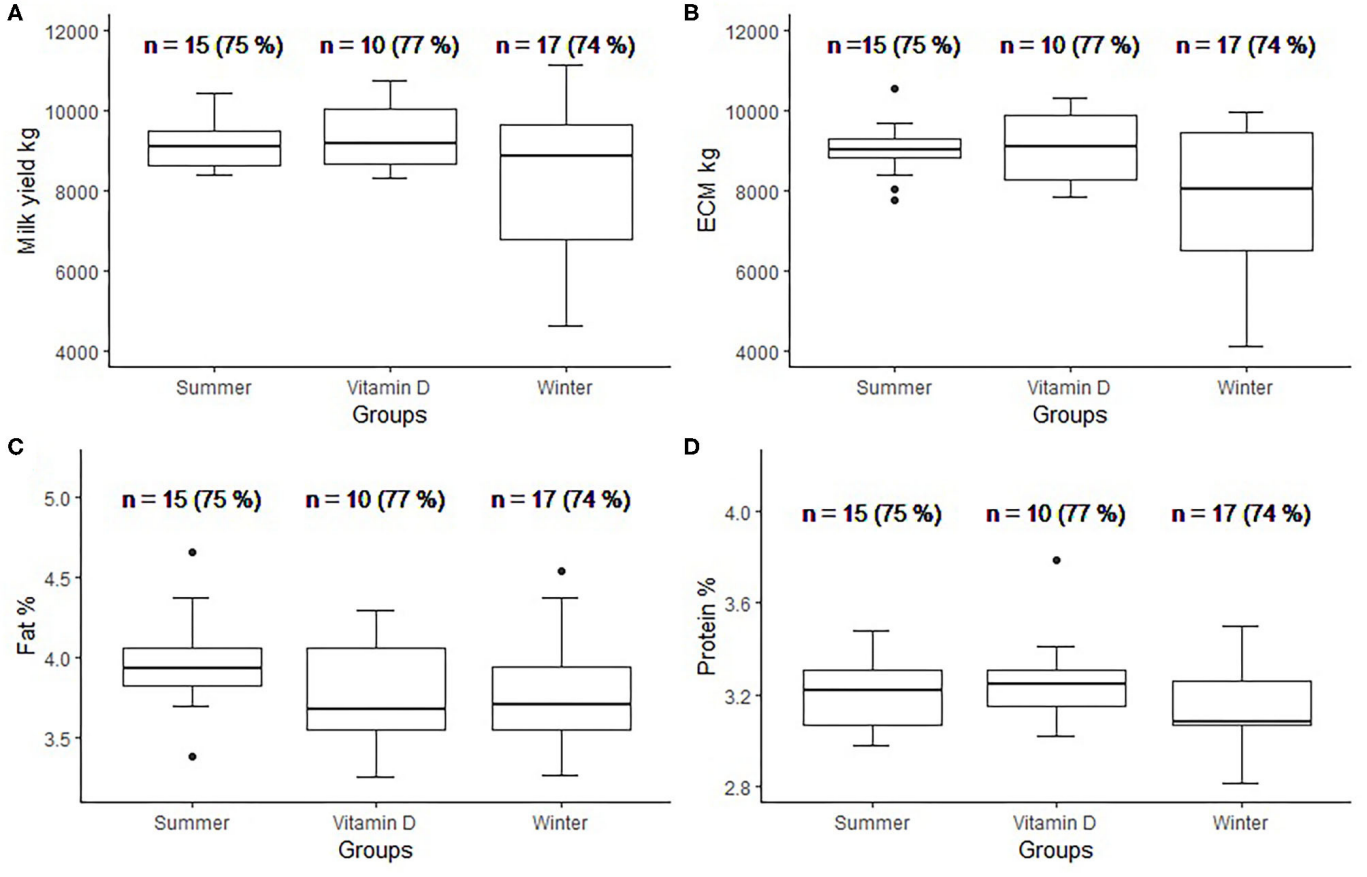
Box plots per group for milk yield **(A)**, Energy corrected milk (ECM) yield **(B)**, fat % **(C)**, and protein % **(D)** in standard lactation (*n* = number (%) of cows that completed standard lactation).

There was no statistically significant difference between groups for milk yield, energy corrected milk (ECM) yield, fat % and protein % in the standard lactation following the closely monitored transition period (Figure 3). The numbers of cows in each group were smaller than at the start of the study because some cows were culled before the end of lactation (the number of culled cows was 5, 3, and 6 for the summer, vitamin D and winter group, respectively). Only complete standard lactations were included in the analysis. In housed groups, the average ECM in vitamin D group was 9,077 kg, while in the winter group the average was 8,001 kg.

### Dynamics of Biochemical Markers

**Figure 4:**
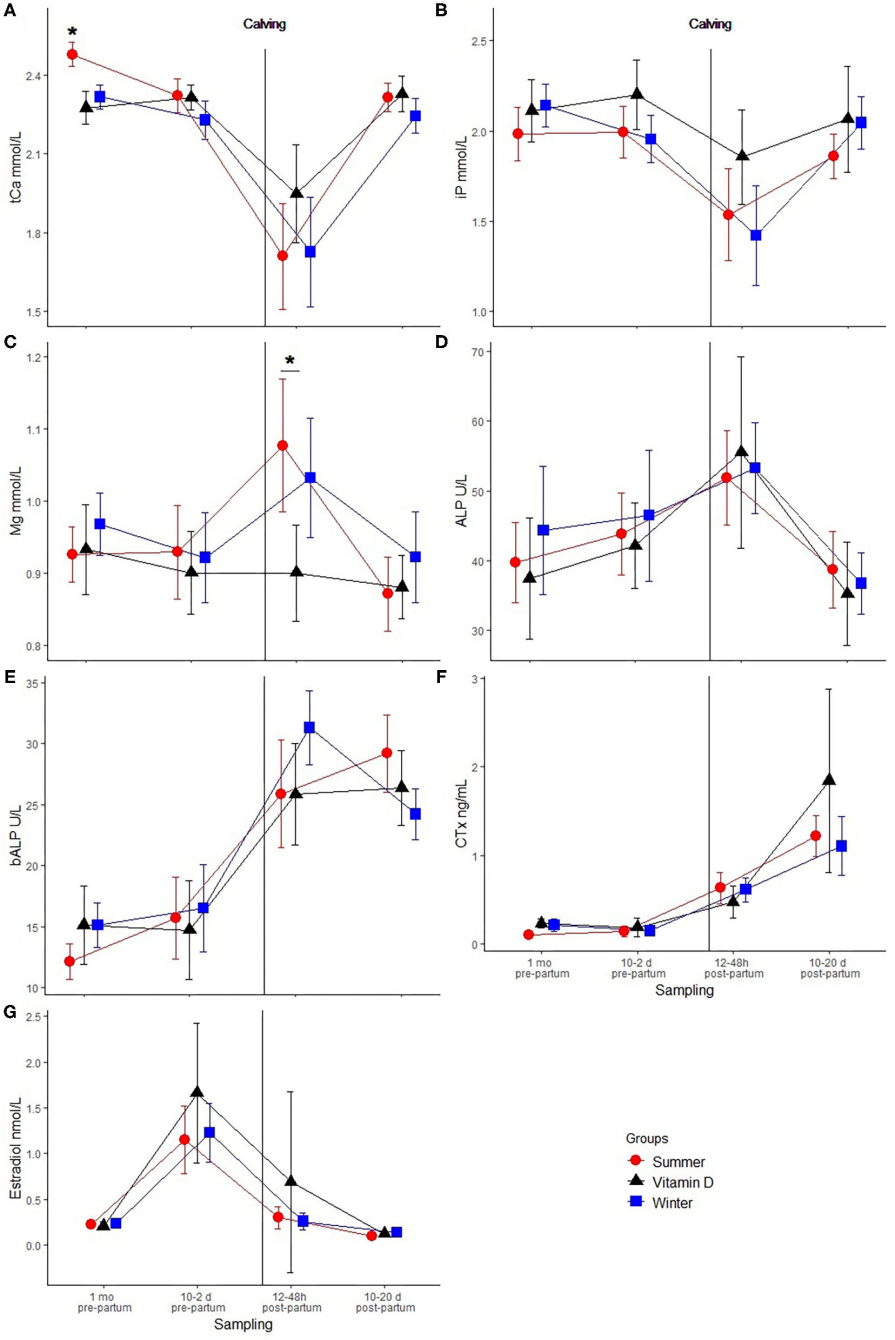
Dynamics of biochemical markers (mean ± 2 SEM) between samplings: total calcium (tCa) mmol/L **(A)**, inorganic phosphates (iP) mmol/L **(B)**, magnesium (Mg) mmol/L **(C)**, alkaline phosphatase (ALP) U/L **(D)**, bone specific alkaline phosphatase (bALP) U/L **(E)**, C-terminal telopeptide of type I collagen (CTx) ng/mL **(F)**, estradiol nmol/L **(G)**. Sampling 1:~1 month pre-partum, 2: 10–2 days pre-partum, 3: 12–48h post-partum and 4: 10–20 days post-partum. The figure also allows the comparison between the summer (*n* = 20), vitamin D (*n* = 13), and winter (*n* = 23) groups. Statistical significance is indicated by: *(*p* < 0.05).

The dynamics of tCa, iP, and Mg are shown in Figures 4A–C. The summer group (pasture) was significantly different in tCa concentrations from the vitamin D and winter groups (housed) in the far-off dry period. Both tCa and iP were lowest after calving but returned to pre-calving levels in fresh cows (Figure 4). The mean (range) values of tCa after calving for the summer, vitamin D and winter groups were 1.71 (0.88–2.35), 1.95 (1.32–2.34), 1.73 (0.57–2.38), respectively. The number (%) of cows with subclinical hypocalcaemia (tCa <2 mmol/L) (14) were 14/20 (70%), 7/13 (54%), 13/23 (57%) for the summer, vitamin D and winter group, respectively. iP mean (range) values after calving for the summer, vitamin D and winter groups were 1.54 (0.37–2.59), 1.86 (1.37–2.81), 1.42 (0.36–2.47), respectively. The number (%) of cows with hypophosphatemia (iP <1.8 mmol/L) (22) were 16/20 (80%), 7/13 (54%) and 17/23 (74%) for the summer, vitamin D and winter group, respectively. Mg had a significant rise after calving, except in the vitamin D group. The vitamin D group had the highest mean values for tCa and iP and the lowest mean Mg value after calving. There was a statistically significant difference in Mg values between the summer and vitamin D group after calving.

The dynamics of ALP, bALP, and CTx can be seen in Figures 4D–F. ALP reached its peak after calving but there was no difference between groups. A rising trend was observed in bALP values except for the winter group that had lower values in early lactation but again there was no significant difference between groups. Similarly, a rising trend in succeeding samplings for CTx was observed, with the winter and vitamin D groups experiencing a slight decrease in the close-up dry period, but there were no significant between-group differences.

Estradiol peaked before calving (Figure 4G). However, we did not detect any between-group differences.

### Correlation Between Estradiol, tCa, iP, bALP, and CTx

Because the data were not normally distributed, we used the Kendall's correlation coefficient. There was no correlation in the first and third samplings. Estradiol was significantly correlated with tCa (τ = −0.25, *p* < 0.05) and bALP (τ = 0.23, *p* < 0.05) 10–2 days before calving. tCa and estradiol were also correlated in early lactation (τ = −0.30, *p* < 0.05). Estradiol in the close-up dry period was not correlated with either tCa, iP, CTx, or bALP after calving.

### The Suitability of Biochemical Bone Markers for Predicting Milk Fever

**Figure 5:**
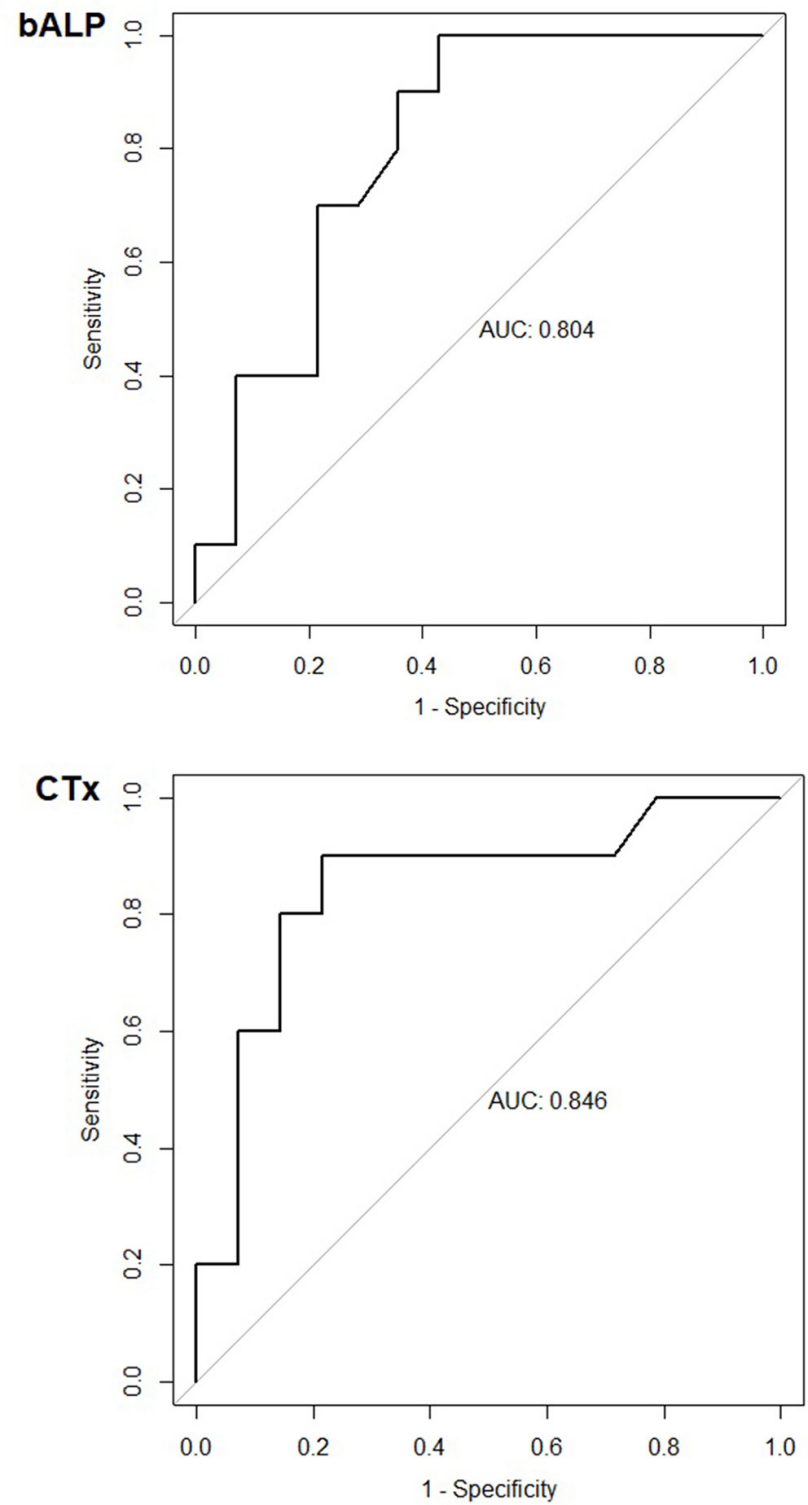
Receiver operating curve (ROC) curve for bone specific alkaline phosphatase (bALP) and C-terminal telopeptide of type I collagen (CTx) for the winter (housed) group. The concentrations of bALP and CTx 10–2 days before calving from cows in the winter (*n* = 23) group were analyzed for their sensitivity and specificity in predicting clinical milk fever after calving. The area under the curves (AUCs) were statistically significantly different from 0.5.

There was no correlation between bALP and CTx 10-2 days before calving on the values of tCa after calving in the summer (pasture) group. However, there was a strong correlation between these markers and tCa in the winter (housed) group. If we combined both groups, we did not find any correlation. Simple linear regression in the winter (housed) group established that bALP at second sampling explains 13.3% (*R*^2^ = 0.133) of the variability of tCa at third sampling (*p* < 0.05). CTx explained 30.7% (*R*^2^ = 0.307) of the variability (*p* < 0.05). Because the correlation between bALP and CTx values and tCa was only found in the winter group we performed the ROC analysis for the winter group only. In the winter (housed) group the diagnostic values using the ROC curve analysis were bALP AUC = 0.804 (95% CI: 0.627–0.980) and CTx AUC = 0.846 (95% CI: 0.675–1.018). Both AUCs were statistically significantly different from 0.5. We determined the optimal cut-off point for sensitivity and specificity of both parameters in the winter (housed) group. The bALP curve had the best ratio at the cut-off point 13.85 U/L with the 90% sensitivity and 64.3% specificity (Figure 5). While CTx had the ratio of 90% sensitivity and 78.6% specificity at the cut-off point 0.149 ng/mL (Figure 5). Logistic regression determined that dairy cows, be it in the winter (housed) or summer (pasture) group, with CTx ≥0.121 ng/mL 10–2 days before calving had a 3.8 times higher chance of getting milk fever.

## Discussion

The study aimed to determine the effect of vitamin D_3_ administration and season (pasture/housed) on selected blood biochemical markers during the transition period and production in the following standard lactation of Holstein-Friesian dairy cows. As far as can be established, no study has looked at the dynamics of biochemical bone markers in transition dairy cattle parenterally (intramuscularly) administered high dose cholecalciferol for the prevention of milk fever or used them as a method for predicting milk fever.

Statistically significantly higher tCa in the far-off dry period in the summer (pasture) group can be explained by the additional vitamin D_3_ cows synthetized while exposed to the sun on pasture, which enhanced intestinal Ca absorption (23).

High dose vitamin D_3_ parenteral administration and season had no statistically significant effect on blood biochemical parameters or milk yield and quality, except for tCa in summer far-off dry period. The administration was not 100% preventative, as two out of the 13 cows developed milk fever, as has been seen in other studies (1, 8). The mechanism of high dose vitamin D_3_ administration does not appear to be related to changes in the studied biochemical bone markers in the transition period according to our results. Kim et al. (22) studied the effect of intramuscular injection of calcitriol to non-pregnant, non-lactating cows and found an increase in osteocalcin, a bone formation marker but not in bALP or bone resorption markers (tartrate-resistant acid phosphatase isoform 5b and hydroxyproline), which is partially in agreement with this study's results. However, in the present study, cholecalciferol was administered within 10 to 2 days before calving, so the physiological condition of tested cows was different. We expected that cow on pasture would synthetize vitamin D_3_ in their skin and thus effect milk fever incidence and blood parameters as was reported by Özçelik et al. (24). But no effect of season (sun exposure) was detected in our study after the far-off dry period. Other studies were also unable to confirm an increase in milk yield or blood minerals in response to vitamin D metabolite supplementation (25). The use of vitamin D and its metabolites to prevent milk fever have been used in many studies with contradicting results. Feeding *Solanum glaucophyllum* a calcinogenic plant containing calcitriol glycosides has increased Ca levels after ethylenediaminetetraacetic acid (EDTA) challenge simulating milk fever (26).

Vitamin D supplementation could have an effect on milk fever incidence by influencing the immune system. Eckel and Ametaj (27) have discussed the positive correlation between endotoxins and a variety of periparturient diseases, milk fever included. Endotoxins can contribute to these diseases by stimulating inflammation or exaggerating the host's immune response (28–30). Vitamin D inhibits proinflammatory interferon-gamma and IL-17 production, by inhibiting antigen-induced CD^+^4 and γδTCR+ T cell proliferation (31). Vitamin D could therefore lower the incidence of milk fever by modulating the immune response, without increasing tCa levels or bone metabolism (31).

tCa and iP concentrations were lowest after calving as established in other studies (6, 9). Prevalence of hypocalcaemia and milk fever was within the expected range for fourth or higher lactation cows fed a diet with DCAD of more than 200 mEq/kg DM in the close-up dry period (1). Mg was highest after calving (32, 33) in all except in the vitamin D group, which showed a decline in Mg concentrations throughout the study. The vitamin D group had the lowest values of Mg and the highest for tCa after calving, which is in-line with the inverse association between Ca and Mg reported by Larsen et al. (34). All measured Mg values in the study were within the reference range for mature cattle, above 0.8 mmol/L (1).

ALP was highest after calving and returned to pre-calving levels 10–20 days after calving as reported by other authors (35). Sato et al. (36) also recorded the lowest ALP levels in dry cows. However, they found ALP was highest in early lactation (8–50 day after calving), while we observed a fall at 10–20 day after calving. Both specific biochemical bone markers increased after calving and were highest 10–20 days after calving (11). Except for bALP in the winter group. There were no significant differences between groups for both markers, which was also the case in a study performed by Liesengang et al. (37). Bone resorption was not increased in cows supplemented with vitamin D_3_.

Estradiol values were highest before calving as was observed in other studies (38). Total Ca was significantly negatively correlated with estradiol levels in the close-up dry period and early lactation. A negative correlation between Ca and estradiol was already documented by Pyörälä et al. (39). The levels of CTx increased after calving after the levels of estradiol decreased. The bone sparing effect of estradiol is well-known (40). Devkota et al. found that osteoclast-mediated bone resorption was activated after calving when estradiol levels in the blood dropped (38). However, we did not observe a statistically significant correlation between estradiol and CTx at any sampling or the correlation of estradiol before and CTx after calving. Results from this study indicate that bone metabolism was increased after calving, in response to high Ca demand in early lactation. Similar conclusions were made by other studies using CTx or other bone resorption markers (11, 37, 41, 42). In a study where biochemical bone markers between healthy and cows with milk fever were compared, higher values of bALP and lower values of CTx were observed in milk fever cows, however, the differences were not statistically significant (33).

The use of bALP and CTx as predictive markers for milk fever showed to be promising, as they were useful for the winter (housed) group. Because there was no correlation between tCa and biochemical bone markers in the summer group, we excluded the group from further calculations. Absence of correlation between tCa and biochemical bone markers could be explained by increased physical activity due to daily access to pasture. This could increase bone turnover thus masking the change resulting from increased Ca demand at calving (43). As cows are not routinely supplemented with high dose vitamin D_3_, the vitamin D group was also excluded from the calculations. In the winter group bALP at the cut-off point 13.85 U/L had 90% sensitivity and 64.3% specificity, while CTx had the ratio of 90% sensitivity and 78.6% specificity at the cut-off point 0.149 ng/mL. A higher sensitivity level was chosen in this study as it is important to detect cows at higher risk of milk fever, rather than miss some because the preventative measures are not harmful to the animal. Because the bALP and CTx are routine automated tests they could be used in practice to detect problematic cows in a herd, which need to be provided with special attention before and after calving. Since most of the milk fever prevention measures are based on feed intervention in the close-up dry period, they require the cows to be separated into multiple groups in the dry period. While this is common in large dairy units it is less practiced on smaller farms. Therefore, the use of this test could be more applicable to smaller herds. The test could also be used to assess the effectiveness of the preventative measures before calving. Some preventive measures like anion salt supplementation can also lower dry matter intake (44). With the use of this test the supplementation could be limited only to cows that really need intervention and thus preserve feed intake in other cows in this critical period. Additionally, at risk cows could be treated with more aggressive or costlier measures at calving to prevent hypocalcaemia or milk fever. Using logistic regression, this study showed cows in summer and winter groups with CTx ≥0.121 ng/mL at 10–2 days pre-partum had a 3.8 times higher chance of getting milk fever, than cow with CTx <0.121 ng/mL in this period. It is counterintuitive that cows with more intense bone resorption in the close-up dry period would have a higher chance of getting milk fever. However, this could mean that these cows had insufficient available blood Ca (ionized Ca) (30) before calving and were compensating with bone resorption. Additionally, the number of 1,25 dihydroxy-cholecalciferol receptors in intestines decreases with age, so cows with high CTx before calving could have been compensating lower Ca resorption from intestines with bone resorption therefore, these cows are also at higher risk for milk fever (45).

Limitations of this study are the relatively small number of cows in each group and a relatively wide time period of blood sampling at each sampling. Vitamin D_3_ and 25-hydroxy vitamin D were not measured in the feed and blood serum, respectively. Methods used to assess biochemical bone markers were not validated for use in cattle and could contribute an analytical error to the study. However, the CTx assay used was also tested in horses and proved to be reliable (46). In our study, a similar pattern of CTx in the transition period compared to other studies using ELISA was observed (11, 31, 35, 36). Roche Diagnostics has used CrossLaps™ One Step ELISA test (Osteometer Meditech Inc., USA) for validation in humans and observed a good correlation (0.883). The same ELISA was also used in a bovine study (47). The assay used in this study uses monoclonal antibodies for the same epitopes as all the ELISAs. Therefore, there shouldn't be a difference in the specificity of detected protein between assays. Further studies are needed, which would include larger samples of cows and also measure total vitamin D in feed, total 25-hydroxy vitamin D in blood serum, and blood ionized Ca which would add another dimension to the interpretation of the mechanism of vitamin D's action in preventing milk fever.

## Conclusion

Despite numerically lowering the incidence of milk fever we cannot conclude that high dose parenteral vitamin D_3_ administration is a viable method for milk fever prevention. Biochemical bone markers in the close-up dry period could be used as an early screening test, which would leave time to intervene in cows at risk of milk fever.

## Data Availability Statement

The raw data supporting the conclusions of this article will be made available by the authors, without undue reservation.

## Ethics Statement

All the procedures on animals were approved by the ethical committee of the Veterinary Administration of Slovenia, approval number: 34401-20/2007/3, and were per the Federation of European Laboratory Animal Science Associations' recommendations. Written informed consent was obtained from the owners for the participation of their animals in this study.

## Author Contributions

JS planning of the study, selection of animals for the study, collection of samples, health monitoring of cows, preparation of samples for the analysis, data collection, writing of the manuscript, and editing of the manuscript. JH writing of the manuscript, statistical analysis, and editing of the manuscript. All authors contributed to the article and approved the submitted version.

## Conflict of Interest

The authors declare that the research was conducted in the absence of any commercial or financial relationships that could be construed as a potential conflict of interest.
